# From Perpetual Wetness to Soil Chemistry: Enumerating Environmental and Physicochemical Factors Favoring *Oncomelania hupensis quadrasi* Snail Presence in the Municipality of Gonzaga, Cagayan, Philippines

**DOI:** 10.3390/tropicalmed9010009

**Published:** 2023-12-29

**Authors:** Daria L. Manalo, Jude Karlo G. Bolivar, Paul Raymund Yap, Ma. Ricci R. Gomez, Zaldy P. Saldo, Mark Joseph M. Espino, Joselito E. Dilig, Raffy Jay C. Fornillos, Shirlyn A. Perez, Regie A. Baga, Louie S. Sunico, Ian Kendrich C. Fontanilla, Lydia R. Leonardo

**Affiliations:** 1 Department of Health, Research Institute for Tropical Medicine, 9002 Research Drive, Filinvest Corporate City, Alabang, Muntinlupa 1781, Philippines; jgbolivar1@up.edu.ph (J.K.G.B.); paulraymundyap@gmail.com (P.R.Y.); maricci.gomez@griffithuni.edu.au (M.R.R.G.);; 2 Institute of Biology, University of the Philippines Diliman, Quezon 1101, Philippinesicfontanilla@up.edu.ph (I.K.C.F.); lrleonardo@up.edu.ph (L.R.L.); 3 Department of Science and Technology, Science Education Institute, Taguig 1631, Philippines; 4 Center for Health and Development Region II, Carig Regional Center, Tuguegarao 3500, Philippines; 5 Local Government of Gonzaga, Gonzaga 3511, Philippines; 6 Office of Research Coordination, University of the East, 2219 C.M. Recto Avenue, Brgy. 404, Zone 41, Sampaloc, Manila 1008, Philippines

**Keywords:** schistosomiasis, *Oncomelania*, environmental factors, physicochemical, snail sites, Cagayan

## Abstract

Snail control to complement mass drug administration is being promoted by the World Health Organization for schistosomiasis control. *Oncomelania hupensis quadrasi*, the snail intermediate host of *Schistosoma japonicum* in the Philippines, has a very focal distribution; thus, scrutinizing baseline data and parameters affecting this distribution is very crucial. In this study in Gonzaga, Cagayan, Philippines, snail habitats were surveyed, and the various factors affecting the existence of the snails were determined. Malacological surveys and the mapping of sites of perpetual wetness in five endemic and five neighboring non-endemic barangays were conducted. Environmental and physicochemical factors were also examined. Maps of both snail and non-snail sites were generated. Of the fifty sites surveyed, *O. h. quadrasi* were found in twelve sites, and two sites yielded snails that were infected with *S. japonicum* cercariae. Factors such as silty loam soil, proximity to a snail site, water ammonia, and soil attributes (organic matter, iron, and pH) are all significantly associated with the presence of snails. In contrast, types of habitats, temperatures, and soil aggregation have no established association with the existence of snails. Mapping snail sites and determining factors favoring snail presence are vital to eliminating snails. These approaches will significantly maximize control impact and minimize wasted efforts and resources, especially in resource-limited schistosomiasis endemic areas.

## 1. Introduction

Schistosomiasis is considered by the World Health Organization (WHO) as a neglected tropical disease that mainly occurs in tropical and subtropical countries. Schistosomiasis contributes to ill health, developmental effects, and socioeconomic effects in areas where the disease is prevalent. It is estimated that the global burden of schistosomiasis is 1.9 million disability-adjusted life years [1]. One species that is still a major public health problem is *Schistosoma japonicum*, despite ongoing mass drug administration. This parasite is endemic in the People’s Republic of China and the Philippines, with small foci in Indonesia. Schistosomiasis japonica is endemic in 12 regions of the country, covering 28 provinces, 14 cities, 189 municipalities, and 2221 barangays [2].

The life cycle of *S. japonicum* needs a snail intermediate host, *Oncomelania hupensis quadrasi*, with a size of 1–5 mm and an adult measuring around 3–5 mm [3] and can be found in areas with perpetual wetness. It is amphibious and thus, can thrive in both water and land; the snail stays in water during the months of breeding and egg-laying and then moves to land for the remaining months of its life [4,5]. Human exposure to the infective larval stages of the parasite is due to contact with water infested with cercariae that emerge from nearby *O. h. quadrasi* snail colonies. As of 2012, potential sources of transmission are 3012 snail-infested bodies of freshwater, of which 80% are in Mindanao, 18% in the Visayas, and only 2% in Luzon [2,6].

Since its discovery in 1906 in the Philippines, the disease has been detected in the eastern and southern regions of the country [7,8]. The first case of schistosomiasis in the northern regions was reported in 2002 in Tapel, a barangay (the smallest administrative subdivision in the Philippines) in Gonzaga, Cagayan, a province at the northeastern edge of the Philippines [6,9]. In 2004, Gonzaga was declared the latest endemic focus of schistosomiasis after the discovery of indigenous cases and infected *O. h. quadrasi* snails, which are the intermediate host in the Philippines. The prevalence of schistosomiasis in Gonzaga, Cagayan is 6%, with a significant proportion consisting in children less than 15 years old [10]. In 2008, the endemicity was further studied in the other three barangays, namely Tapel, Magrafil, and Sta. Maria [6]. According to Gonzaga LGU, and the DOH II, in 2018, two additional barangays, Cabiraoan and Sta. Cruz were declared new endemic areas for schistosomiasis with the discovery of *O. hupensis quadrasi*, infected residents, and ongoing transmission. New snail sites have also been discovered in China despite ongoing snail control, indicating the emergence of new habitats for spreading snails [4].

In the Philippines, schistosomiasis prevention and control strategies include case finding, treatment, environmental sanitation, snail mapping and control, health promotion, surveillance and monitoring, quality control, and networking and linkages [11]. However, only mass treatment is being implemented regularly, which is insufficient to control the disease. The WHO now recommends snail control to complement mass drug administration as one of the recent strategies to ultimately eliminate the disease [12,13,14]. The more aggressive combined strategies (community-wide MDA and snail control) have proven to be the most effective in reducing both prevalence and infection intensity [15,16]. However, snail sites are very focal, which explains the focal transmission of schistosomiasis.

This study aimed to survey all sites with perpetual wetness in the five endemic and non-endemic neighboring barangays in Gonzaga, Cagayan to identify and map both snail and non-snail sites. This study hopes to identify the key factors that can be used to distinguish a snail site from a non-snail site. The protocols generated from this study can potentially be scaled up to include other endemic areas, resulting in more focused control, and prevention strategies in areas of active and intense transmission.

## 2. Methods

### 2.1. Study Area

**Figure 1:**
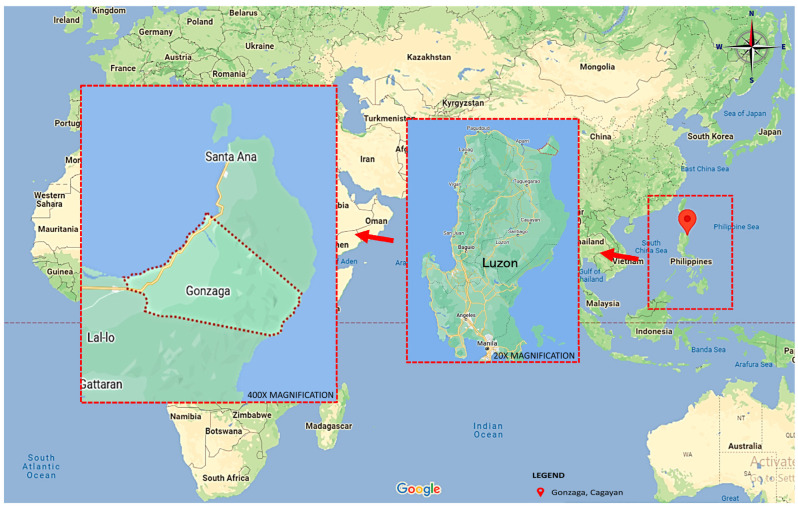
Geographical location of the study area relative to the Philippines.

Gonzaga (Figure 1) is a municipality located at the northeastern tip of the province of Cagayan, 125 km from the provincial capital of Tuguegarao City and 607 km from Manila. It is politically subdivided into 25 barangays. Gonzaga has a total land area of 56,743 hectares, with the majority remaining undeveloped. There are 11 coastal barangays, which include a total of 139 hectares of beaches, 69 hectares of mangrove forests, and 348 hectares of coral reefs. According to DOH Cagayan, as of 2018, there were five schistosomiasis-confirmed endemic barangays, Tapel, Magrafil, Santa Maria, Cabiraoan, and Santa Cruz. This study covered the five current endemic barangays as well as five neighboring non-endemic barangays, namely Santa Clara, Calayan, Baua, Ipil, and San Jose.

### 2.2. Malacological Survey

All sites with perpetual wetness were included in an intensive survey. Each site was divided into four sections, and a snail collector proficient in identifying and collecting *O. h. quadrasi* was assigned to each section. In linear sites, the length and width of collection areas were measured to equally divide the survey area. On the other hand, non-linear sites were divided into quadrants, and the total area of each quadrant was measured. The snail collectors were given a 5-min time limit to cover a 4–5 m^2^ study area.

*Oncomelania* snails were identified as operculated; their size ranges from 1 to 5 mm, with a smooth shell, acuminate, dextral, light to brown to even partly or wholly black. The shell has at most six whorls, with the spire eroded most of the time. The operculum is ovate, thin, transparent, and with a large eccentric nucleus. The aperture is also ovate corresponding to the shape of the operculum and measuring 1.60 to 2.04 by 1.00 to 1.30 mm [5,17]. A site is identified as a snail site if *O. h. quadrasi* is found and as a non-snail site if otherwise. For further analysis, the number of snails collected was recorded.

Collected snails were placed in paper envelopes and then transported to the laboratory, where they were wiped with paper towels to remove the attached soil debris. The specimens were then placed on clean sheets of filter paper and air-dried.

### 2.3. The Cercarial Infection Rate of the Snails

Three equidistant aliquots of distilled water (approximately a droplet) were placed on a clean glass slide, with one *O. h. quadrasi* snail placed in each aliquot. The snails were gently crushed by pressing another clean slide on top of them. Each crushed snail was examined individually using a stereomicroscope (10×) (Nikon, C-LEDS 100, Tokyo, Japan) to determine *S. japonicum* cercaria infection. Snails were classified as infected if they showed the characteristic schistosome bifurcated cercariae only and not sporocysts. The infection rate was calculated by dividing the number of infected snails by the total number of collected snails and then multiplying the result by 100.

$$Infection\;Rate\left(IR\right)=\frac{no.of\;infected\;snail}{total\;number\;of\;collected\;snails}\times 100\%$$

### 2.4. Mapping of Snail Sites in Gonzaga, Cagayan

The GPS coordinates of each surveyed site were recorded and encoded using QGIS software (QGIS 2.8, Wien). Distance and elevation values were extracted from the GPS (Garmin GPSMAP 78) data. The most recent irrigation map of Gonzaga, Cagayan was obtained from the National Irrigation Administration (NIA) of Region II. The map was updated by conducting an actual survey of irrigation canals, water networks, and tributaries, as well as network leaks and wastewater from rice fields. Barangay officials assisted in locating the source of the leaks and wastewater. All GPS coordinates of the identified water systems were recorded.

### 2.5. Environmental and Physicochemical Characteristics

In all surveyed sites, environmental parameters were measured. Different habitat forms, such as creeks, streams, ponds, waterlogged areas, irrigation canals, river tributaries and pockets, swamps, leaks from irrigation, springs, and a combination of two or more of these habitats, were identified and described. The type of soil was also analyzed.

### 2.6. Physicochemical Characteristics of the Soil and Water

The surface temperatures of both water and soil were measured. The pH of the water was also taken. The water was collected early in the morning and transported at a temperature between 2 °C and 8 °C immediately to the laboratory to meet the required storage conditions. The Bureau of Fisheries and Aquatic Resources (BFAR) Region 2 facilitated the physicochemical evaluation of one liter of water within 4–5 h after collection. The water was tested for physicochemical characteristics such as dissolved oxygen, biological oxygen demand, ammonium nitrogen, nitrite-nitrogen, and carbon dioxide using the LaMotte Freshwater Aquaculture Test Kit Model AQ-2 (LaMotte Co., Chestertown, Maryland, USA). For the soil analysis, approximately 2 kg (kg) of soil were collected from every site. The soil samples were air-dried, and 1 kg of dried soil collected per site was submitted to the Soils Laboratory of the Integrated Laboratory of the Department of Agriculture Region 2 for testing. The soil type or texture was investigated by using the finger method as described by the *Soil Survey Manual* (USDA 2017) [18]. The pH and phosphorus levels of the soil were determined using potentiometric tests and Olsen tests, respectively. The organic matter (nitrogen) of the soil was assessed using the Walkley and Black spectrophotometric methods. The potassium content was measured using the cold sulfuric extraction method. The zinc, copper, manganese, and iron contents of the soil were analyzed using the diethylenetriaminepentaacetic acid (DTPA) micronutrient extraction method. Both the BFAR and Soils Laboratories are under the Department of Agriculture and thus adhere to the standards of ISO/IEC 17025 to maintain their quality management system demonstrating competent operation and generating valid results.

### 2.7. Statistical Analysis

A descriptive analysis of the physicochemical features of the soil, water, topography, and presence of vegetation was conducted. Categorical variables such as soil type, habitat, and species of plants were described using frequency and percentage, while continuous variables such as temperature, elevation, soil, and water chemistry were described using mean and standard deviation, median, and range. Fisher’s exact test was used to determine if there are associations between habitat and soil type, and the presence of snails. The odds ratio with a 95% confidence level is the measure of association. A t-test was used to compare the soil and water characteristics and the distance to the nearest snail sites in areas with and without snails. A *p*-value of less than 0.05 was used to designate statistical significance.

## 3. Results

### 3.1. Malacological Survey and Mapping

**Figure 2:**
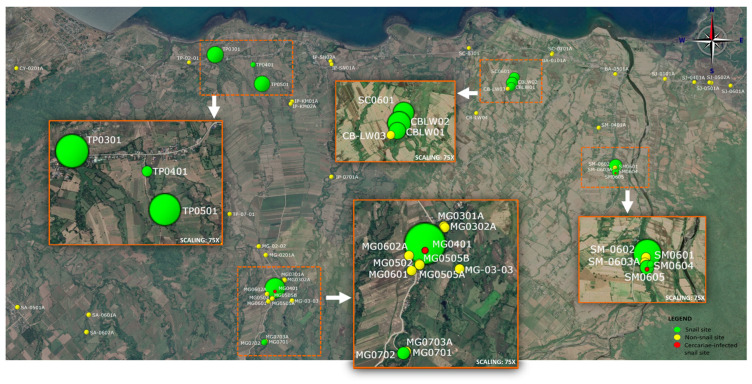
Map of the 50 surveyed snail sites from 5 endemic and 5 non-endemic neighboring barangays of Gonzaga, Cagayan. All snail sites were assigned codes represented by two-letter shortcuts of the name of the barangay, followed by a 2-digit Purok (district) number, then the last two digits signifying which specific site. Two-letter barangay names are as follows: Endemic: CB—Cabiraoan, MG—Magrafil, SC—Sta Cruz, SM—Sta Maria, and TP—Tapel; Non-endemic: BA—Baua, CY—Calayan, IP—Ipil, SJ—San Jose and SA—Santa Clara. The number of snails collected is represented by the green circumference of each point, a larger circumference signifies a higher number of snails. Red dots represent sites with snails infected with *S. japonicum* cercariae. Non-snail sites surveyed are colored yellow.

Fifty sites were surveyed for *S. japonicum* in Gonzaga, Cagayan, Philippines in five endemic and five neighboring barangays. The actual snail sites are still the same sites identified in 2018, and no *O. h. quadrasi* snails were found in non-endemic barangays (Table 1). Out of the 50 sites, there are 12 (24%) actual snail sites with the confirmed presence of *O. h. quadrasi* snails; these are all highlighted in Figure 2. Of these, only two sites yielded snails that were infected by *S. japonicum* cercariae. Two of the snails infected by cercariae were from Sta Maria (SM0605), and one was from Magrafil (MG0401). Snail infection rates from these two snail sites were noted as 3.57% and 0.32%, respectively.

A total of 1439 snails were collected, 59% of which were juveniles (less than 3 mm in size), with an average snail density of one snail per two square meters (m^2^), and an average cercarial infection rate of 0.2%. The area of snail sites ranges from 15 m^2^ to 1540 m^2^, with a total of 2875 m^2^ of snail sites in the five endemic barangays.

The distance of the surveyed sites to the site that harbors snails was found to be significantly different in areas with snails and those without snails (*p*-value = 0.002) (Table 2). Results indicate that the average distance between snail sites is 488.38 m. On the other hand, sites without snails have an average distance of 1529.44 m away from the nearest site with snails. The elevation was found to have no significant difference between sites with snails and those without.

### 3.2. Environmental Parameters

**Figure 3:**
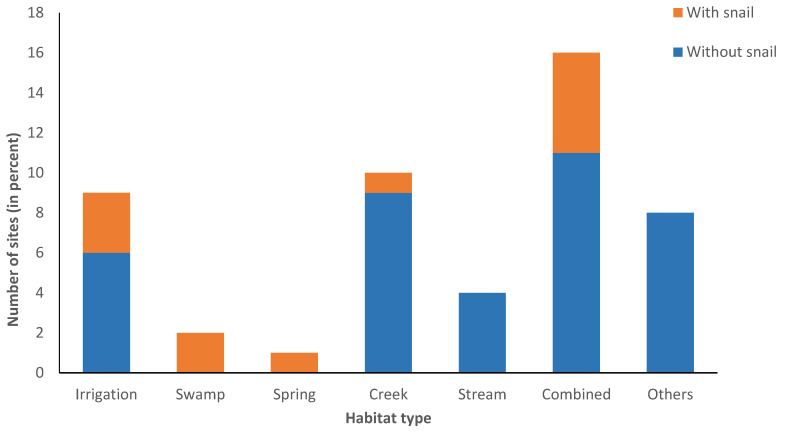
Habitat type and presence of snail.

**Figure 4:**
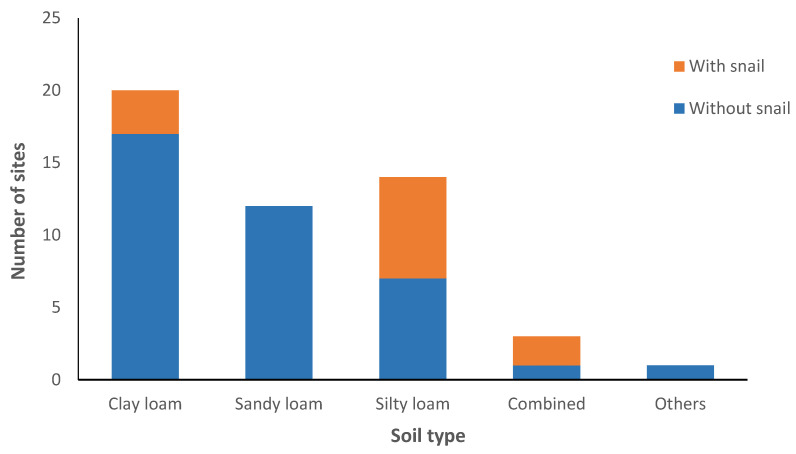
Type of soil and the presence of snails.

Of the 50 sites surveyed, 16 sites have combined habitats (a mixture of various habitats in one site) (Figure 3), and 31.25% of these combined habitats were found to have snails. All spring and swamp habitats harbored *O. h. quadrasi* snails, while all streams were negative for snails. However, there was no link between the type of habitat (stream, spring, creek, irrigation, swamp) and the presence or absence of snails. The presence of snails, however, was linked to the type of soil (Figure 4). Most of the soil types surveyed are clay loam (36.73%). Only the silt loam soil has a statistically significant association with the presence of snails (*p* = 0.029). No snails were found in the sandy soil, and a significant association with the absence of snails was established (*p* = 0.024).

### 3.3. Physicochemical Characteristics of Water and Soil

The pH, iron, and organic matter (OM) content of soil were found to be statistically different in areas with snails and those without (Table 3). Snails were found in soil with slightly acidic pH (5.72 + 0.57), high OM (3.27 + 0.88), and high iron content (110.72 + 65.03). Among the water characteristics, low NH_3_ levels (0.18 + 0.06) were associated with snail presence (*p* = 0.0458). There was no significant difference in soil or water temperature between snail and non-snail sites (Table 3).

## 4. Discussion

The results of the study confirmed that barangays Cabiraoan, Magrafil, Sta. Cruz, Sta. Maria and Tapel have *O. h. quadrasi* snail sites. The neighboring barangays, on the other hand, were found to still be free of the snails. The snail sites in each village or barangay are very focal and they occur in clumps [3,19,20,21]. One snail present in an area does not suggest the presence of a snail colony at that site because this snail might not have been originally there. The snail could have been carried by water currents or released from previously dried mud on the skin of animals like carabaos, human footwear, or some agricultural tools. The snail sites can be found in a very limited and confined area, as shown on the map, consistent with previous findings such as those in Samar, Philippines, where snail colony distribution is limited [19]. This implies that snails have optimal environmental requirements to establish a snail colony [5,22,23,24,25]. *O. h. quadrasi* has been observed to inhabit multiple, varying habitats such as swamps, along margins of sluggish streams neighboring spring outlets, roads, irrigation and borrow ditches, abandoned rice fields, creeks, and huge waterlogged areas, as well as pockets of medium and large rivers [19,26]. However, in this study, the surveyed snail sites have not been linked to a particular habitat. Thus, it is hard to conclude from this study that swampy areas or spring outlets are right away snail habitats but are simply considered potential habitats that can become actual habitats if given optimal conditions.

A very distinct feature of the habitat of *O. h. quadrasi* is perpetual wetness [5,26,27,28,29]. The snail is amphibious, staying in water during breeding months and egg-laying and moving to land for the rest of its life [5]. The snails primarily require water for their development and survival. These snails may be found in various aquatic habitats, such as ponds, rivers, streams, marshes, and other areas with ample water availability. Snails often lay their eggs in water or moist areas near water bodies. Water is essential for hatching, and the early development of snail hatchlings. Without a suitable aquatic environment, the reproduction of *Oncomelania* snails may be severely impacted. Even adult *Oncomelania* snails likely need a consistently moist environment. They may spend part of their lives submerged in water and part of it in damp, terrestrial areas adjacent to water bodies [27,30].

In this study, the snails’ soil preference is silt loam, which is consistent with the findings of Nihei [31]. Loam and silty soils contain organic matter from which the snail obtains its nutrients. This study proves that high organic matter in the soil favors snail survival as previously shown by Calata et al. [3]. The sandy soil was also shown to be unfavorable for the presence of snails because coarse soil prevents them from crawling [28] and does not favor breeding [32].

Madsen et al. [20] noted that most of the physicochemical characteristics of soil and water do not have any correlation with the snail’s presence. Soil chemistry was observed to have no effect on the distribution of *Oncomelania* snails in Palo in Leyte Island, to the northeast of Bohol, Philippines, and pH was not considered a factor limiting the distribution of *Oncomelania* spp. because snails can tolerate a wide range of pH values [5,27]. However, in this study, soil pH and the presence of organic matter and iron are significant factors in the presence of the snail. The finding of Calata et al. [3] that soil pH affects the presence of snails in Gonzaga, Cagayan was confirmed in this study. Soil pH can also affect the availability of nutrients, and the solubility of minerals, which can indirectly influence the snail population. A slightly acidic pH is tolerable for snails; however, an acidic pH of less than 4.8 can result in erosion of the shell, and eventually death. Moreover, acidic pH can soften the shell and retard snail growth. The soil may become slightly acidic because of the decaying organic matter. The productivity of *O. quadrasi* is low when the pH of the substrate is >9 or <4.6 [5,32]. Furthermore, it was noted that alkaline water (pH = 7.6) is essential for the natural release of cercariae of *S. japonicum* from *O. h. quadrasi* in Leyte, Philippines [27,33]. In addition, the organic content and degree of pollution are of primary importance in the distribution of *O. h. quadrasi*. The organic matter derived from plants, animals, and human excreta serves as food for snails, and fertilizer for unicellular algae that constitute important food for young snails and provide growth factors that trigger snail reproduction [34]. Additionally, organic matter can help retain moisture in the soil, creating a more suitable habitat for snails. Aside from organic matter, *Oncomelania* requires iron for its nutrition. The presence of iron in the soil may affect the abundance and distribution of snails, especially if it influences the types of plants or algae available as food for snails.

The temperature of both water and soil is a considerable factor in snail distribution in China [24,25,31,35,36,37]. In the Philippines, the temperature in some areas affects the growth of snails [5,31], but not their presence [3]. The suitable temperature for mating is 15–20 °C, extreme temperatures outside of this range may disrupt or inhibit their mating activities, while the most suitable temperature for its spawning is 20–25 °C. This temperature range may provide optimal conditions for the development and survival of their eggs [24]. This study further confirms that the temperatures of the soil and water at each site in Gonzaga, Cagayan, do not determine the presence of snails since these organisms can tolerate a wide range of temperatures. Snails prefer shady areas [5]; thus, when the temperature is high, the snails migrate beneath the water surface, especially underneath the vegetation present, and congregate under the shade provided by overhanging trees and shrubs. In contrast, during the early morning and late afternoon when the temperature is cool, snails can be found on leaves and branches of vegetation. In this study, temperatures were not taken at a particular time of the day; thus, varying temperatures were obtained. Sites surveyed in the middle of the day would have very high temperatures, and for sites surveyed early in the morning, the temperature would have been lower; these data do not correlate with the presence or absence of snails.

Another factor to consider in the presence of snails is elevation. In China, the elevation of infested marshland with *Oncomelania* ranged from 9 m to 16 m [38,39] while the elevation in infested mountainous areas is below 2300 m [40]. In Gonzaga, Cagayan, the snail sites are all in the marshland where the elevation ranges from 11 to 173 m. Aside from elevation, distance from the nearest snail site is a factor to consider. The proximity of each site to each other implies that the snails can be transported by animals, humans, or water currents. In China, the variability of the distribution pattern of live snails mainly occurs within the village boundary [41]. Environmental conditions can be a key idea to briefly discuss why snails are present or absent at a particular surveyed site.

These factors that affect the presence of snails are complex and have been known to exert combined and different effects in different regions. The ecological and environmental factors indicate that the distribution of snail habitats is area specific. Although control strategies can be universal, one still needs to apply very specific strategies that apply to Gonzaga, Cagayan.

The findings of the study, which show the factors that favor and disfavor snail presence, can be used to eliminate or prevent the establishment of snail habitats. Removing the water source is the ultimate strategy to eliminate snails, but due to the improbability at most sites, the water may be redirected to areas unfavorable for snails. Non-snail sites with perpetual wetness, swampy conditions, and near springs with silt loam soil should be surveyed regularly to avoid the emergence of snails. Water should be drained, and the embankments should be reinforced with concrete cement if possible. Clearance of vegetation and the application of chemical molluscicides (Niclosamide and Phebrol) for the control of *O. h. quadrasi* have not yet yielded satisfactory results in certain snail colonies; therefore, they are not recommended for snail control [29]. In contrast, the environmental survey performed at the respective sites can initiate modification of the identified conditions or factors to inhibit snail survival.

## 5. Conclusions

Chemotherapy will not be enough to achieve elimination of the disease in 2030, hence the recommendation to include snail control. Identifying the snail sites and knowing the corresponding environmental requirements for the snails’ survival and growth are significant considerations in achieving snail control. Some prevention and control methods that are applicable in one endemic area may not be applicable in another; hence, environmental diagnostics are needed to determine exactly where the snail sites are and what environmental factors perpetuate these sites. The outcome of the study was limited to snail presence, absence, and association with environmental factors. The effects of the co-inhabitants of other snails, vegetation density, and anthropization of habitats to the presence of snails can be avenues for future research on *Oncomelania*. While this study provides initial strategies for environmental diagnostics, it is recommended to employ modern molecular biology techniques such as polymerase chain reaction (PCR) and loop-mediated isothermal amplification (LAMP) to obtain more accurate and faster results [42], while also reducing the harm to the environment. In addition, these faster platforms could be a potential tool to investigate other snail species in the area and to determine whether they can also influence the presence or absence of *O. h. quadrasi*.

## Figures and Tables

**Table 1:** Total number of snails collected, cercariae-infected snails, and snail infection rate from five endemic barangays surveyed in Gonzaga, Cagayan.

Endemic Barangay	Surveyed Site	Collected Snails per Site	Total Number of Snails per Barangay	Number of Cercariae-Infected Snails per Site	Snail Infection Rate (%) per Site
Cabiraoan	CBLW01	85	221	0	0.00
	CBLW02	136			
	CBLW03 CBLW04	0			
Magrafil	MG0401	308	320	1	0.32
	MG0505A	1		0	0.00
	MG0702	11			
	MG0201A MG0202 MG0301A MG0302A MG0303 MG0502 MG0503 MG0505B MG0601 MG0602A MG0701 MG0703A	0			
Sta Cruz	SC0601	102	102	0	0.00
	SC-0301 SC-0701A				
Sta Maria	SM0605	56	205	2	3.57
	SM0601	144		0	0.00
	SM0604	5			
	SM-0401A SM-0602 SM-0603A	0			
Tapel	TP0301	285	591	0	0.00
	TP0401	11			
	TP0501	295			
	TP0101 TP-02-01 TP-07-01	0			

**Table 2:** Mean elevation and distance of the surveyed sites to a known snail site.

Factors	Non-Snail Sites		Snail Sites		*p*-Value
	Mean	SD	Mean	SD	
Distance (m)	1529.44	1379.44	488.38	518.43	0.002 *
Elevation/inclination (m)	75.94	68.93	54.91	62.00	0.113

* *p*-value < 0.05 is statistically significant.

**Table 3:** Physicochemical characteristics of soil samples and collected water.

**a. Soil Samples**					
**Physicochemical Parameters**	**Non-Snail Sites**		**Snail Sites**		***p*-Value**
	**Mean**	**SD**	**Mean**	**SD**	
Temperature (°C)	29.40	2.63	28.90	2.94	0.5845
pH	6.20	0.66	5.72	0.57	0.0269 *
(Organic matter) OM	2.46	1.20	3.27	0.88	0.0183 *
phosphorus	19.91	18.02	20.80	16.32	0.8802
K	318.97	194.37	349.17	106.92	0.5999
Zn	3.96	7.79	3.10	3.39	0.7152
Cu	5.32	3.02	5.50	2.29	0.8551
Mn	36.66	16.87	35.78	18.02	0.8784
Fe	65.02	36.34	110.72	65.03	0.0035 *
**b. Collected Water**					
* Physicochemical Parameters	**Non-Snail Sites**		**Snail Sites**		***p*-Value**
	**Mean**	**SD**	**Mean**	**SD**	
Temperature (°C)	28.19	2.55	28.38	1.61	0.8143
pH	7.13	0.77	7.09	0.55	0.8774
dissolved Oxygen	4.14	1.00	3.93	1.36	0.5883
NH_3_	0.35	0.46	0.18	0.06	0.0458 *
NO_2_	0.027	0.025	0.032	0.025	0.5950
CO_2_	13.34	10.22	23.45	36.38	0.3830
Total Hardness	55.83	30.96	51.27	18.56	0.6474

* *p*-value < 0.05 is statistically significant, *n* = 50 sampling sites.

## Data Availability

The datasets used and analyzed during the current study are available from the corresponding author upon reasonable request.
